# Effects of a Nutritionally Balanced Between-Meal Food Product on Brain Function in Older Adults: Protocol for a Randomized Controlled Trial (the MiniMeal Study)

**DOI:** 10.2196/94648

**Published:** 2026-09-17

**Authors:** Lina Tingö, Jort Veen, Pernilla Andersson, Myrto Chatzopoulou, Julia Rode, Kristina Andersson, Işılay Kaya, Ana Rascón, Peter Edholm, Robert Brummer, Cecilia Bergh, Ashley N Hutchinson

**Affiliations:** 1 Nutrition-Gut-Brain Interactions Research Center School of Medical Sciences, Faculty of Medicine and Health Örebro University Örebro Sweden; 2 Medicine and Caring Sciences Department of Health Linköping University Linköping Sweden; 3 Food and Health Centre Örebro University Örebro Sweden; 4 School of Health Sciences, Faculty of Medicine and Health Örebro University Örebro Sweden; 5 Glucanova AB Lund Sweden; 6 Department of Process and Life Science Engineering Division of Food and Pharma Lund University Lund Sweden; 7 Department of Environmental and Bioscience Halmstad University Halmstad Sweden; 8 Clinical Epidemiology and Biostatistics School of Medical Sciences, Faculty of Medicine and Health Örebro University Örebro Sweden

**Keywords:** nutrition, inflammation, cognitive function, brain activity, healthy aging, chickpeas, oats, gut microbiota

## Abstract

**Background:**

Increasing evidence suggests that diet influences gut microbiota composition and systemic inflammation, which in turn may affect brain health, cognitive function, and mood regulation. Elevated levels of systemic inflammation have been associated with reduced resting-state functional connectivity within the default mode network, a pattern linked to poorer cognitive performance in older adults. Dietary interventions incorporating anti-inflammatory foods, such as oat bran and chickpeas, may help attenuate low-grade inflammation and promote healthier dietary behaviors in aging populations.

**Objective:**

This study protocol describes a randomized controlled trial designed to investigate the effect of healthy between-meal products on brain activity in older adults.

**Methods:**

The MiniMeal study is a 9-week, 3-arm randomized controlled trial comprising a blinded, 2-arm intervention and a nonblinded, no-intervention comparison group. A total of 114 community-dwelling men and women aged ≥70 years (38 per group) will be randomized to 1 of 3 study arms. Participants in the intervention group will consume the MiniMeal products, while those in the reference or control group will receive reference or control products. In both intervention arms, products will be consumed twice daily, either replacing or supplementing habitual morning, afternoon, or evening between-meal occasions. All other dietary habits will remain unchanged. Participants in the no-intervention group will maintain their habitual diet throughout the study period. The primary outcome is functional brain activity, assessed using functional magnetic resonance imaging during the n-back task, comparing the intervention and reference groups. Secondary outcomes include comparisons with the no-intervention group and assessment of other neural measures, biomarkers in blood, body composition, physical function, and self-reported well-being.

**Results:**

Ethics approval was obtained from the Swedish Ethical Review Authority (Etikprövningsmyndigheten) on February 20, 2024. Participant recruitment was completed on December 18, 2025. Data collection was completed by February 27, 2026. Data analysis is ongoing since January 7, 2026. A total of 114 participants were enrolled, of whom, 106 completed all 6 study visits.

**Conclusions:**

This study will provide insights into whether nutritious between-meal products influence brain function in older adults and will explore potential underlying biological mechanisms. The findings are expected to contribute to a better understanding of the relationship between dietary habits and cognitive health in older adults and may inform future research on diet-based strategies to support in healthy aging.

**Trial Registration:**

ClinicalTrials.gov NCT06353984; https://clinicaltrials.gov/study/NCT06353984

**International Registered Report Identifier (IRRID):**

DERR1-10.2196/94648

## Introduction

### Background and Rationale

Population aging is a worldwide phenomenon that constitutes a major public health challenge, as it is linked to increased health care demands for age-related disabilities, such as cognitive impairment [1]. Evidence suggests that the gut-brain axis (GBA) is important for brain health, as well as for cognition and mood regulation [2,3]. Furthermore, dysregulation of this axis is associated with neurodegenerative and neuropsychiatric disorders including dementia, Parkinson disease, and depression [4-6].

Certain features of the GBA also seem to be intricately linked to the function of the immune system [7,8]. For example, gut microbiota aberrations may trigger low-grade systemic inflammation [3,9], a common condition among older adults, which has been suggested to impact brain function and contribute to cognitive decline [10,11]. For example, it has been shown that higher levels of systemic inflammation are specifically associated with lower resting state functional connectivity between brain regions in the default mode network (DMN), a network whose altered functional connectivity has been associated with reduced cognition among older adults [12,13]. Hence, low-grade systemic inflammation and age-related changes of the gut microbiota may be seen as critical factors in the development of cognitive decline in older adults.

Increasing evidence suggests that diet may affect both the gut microbiota and systemic inflammation [9,14]. During aging, appetite, dietary requirements, and changes in dietary behaviors increase the risk of malnourishment [15]. Malnourishment is accompanied with decreases in muscle mass, muscle strength, and bone mineral density, which lowers physical function and increases the risk for frailty [16-18]. Frailty is also coupled with impairments in the immune system and cognitive decline [19,20]. Taken together, strategies to change dietary behaviors in (especially) vulnerable older adults are warranted.

For older adults failing to meet their daily energy requirements, between-meals snacks can be an effective strategy to promote adequate caloric and nutritional intake [21]. However, the readily available commercial options often consist of a rather unhealthy nutritional profile, containing low quality carbohydrates and unhealthy fats and lacking dietary fiber and proteins. Diets high in glucose and fat but low in protein, fiber, and certain micronutrients may contribute to the development of low-grade inflammation [22], whereas anti-inflammatory diets including Mediterranean diets [23,24], the Nordic diet [25], and diets containing prebiotic components [26-28] seem to exert opposite effects. Such anti-inflammatory dietary patterns have also been linked to improved cognitive function in older individuals [29].

Taking these aspects into consideration, new, drinkable, between-meal products (MiniMeal) were designed based on bioprocessed oat bran and chickpea, providing a well-balanced nutritional profile with high-quality protein and mixed-origin soluble and insoluble dietary fiber. Several previous studies have demonstrated the positive impact of oats on the gut microbiota [30,31], satiety [32], blood pressure [33,34], cholesterol, and glucose regulation (European Food Safety Authority Panel on Dietetic Products, Nutrition, and Allergies). Furthermore, oats have been shown to have anti-inflammatory properties [35,36] and improve cognitive function related to both long- and short-term memory [31]. Chickpeas have been shown to reduce total and low-density lipoprotein cholesterol [37,38], improve homeostatic model assessment for insulin resistance index [37], lower inflammation [1,2], and may modulate the gut microbiota [3-5]. Moreover, evidence from preclinical studies supports the potential neuroprotective role of chickpeas, due to their antioxidant and anticholinergic properties [39,40].

### Objectives

The nutritionally well-balanced MiniMeal between-meal products evaluated in this project (a soup and a fruit drink) contain ingredients with the potential to impact all the above-described health outcomes. By targeting risk factors related to malnutrition, gut-microbiota alterations, and systemic inflammation, these products may promote favorable changes in functional brain activity and cognitive performance. This paper outlines the study protocol for a randomized controlled trial in older adults, designed to evaluate the effects of the MiniMeal products on brain activity during a cognitive task as the primary outcome.

## Methods

### Study Design

The MiniMeal study is a 3-arm, randomized controlled trial that includes a blinded, 2-armed intervention and a nonblinded, no-intervention comparative arm.

### Study Setting

The MiniMeal study will be conducted at Örebro University School of Medical Sciences and the Center for Experimental and Biomedical Imaging in Örebro (CEBIO) at the Örebro University Hospital.

### Eligibility Criteria

Inclusion and exclusion criteria are found in Textbox 1.

**Inclusion and exclusion criteria for participant eligibility in the MiniMeal randomized controlled trial.**

**Inclusion criteria**

Demographics

- Signed informed consent prior to any study-related procedure
- Age ≥70 years
- BMI range 18.5-31.9 at the screening visit

Other

- Will to abstain from medication known to alter gastrointestinal function or inflammatory status after being included and during the study
- Willingness to pick up study products and eat the products according to the instructions each day

**Exclusion criteria**

Comorbidities

- Diagnosis of type 1 and/or type 2 diabetes
- Diagnosed inflammatory bowel disease (IBD)
- Current diagnosis of psychiatric diseases or syndromes
- Current diagnosis of neurodegenerative disease
- Any condition that could interfere with intestinal barrier function (eg, gluten sensitivity, lactose intolerance, celiac disease, irritable bowel syndrome, and IBD) as decided by the principal investigators’ (ANH and CB) discretion
- History of complicated gastrointestinal surgery
- Cerebral bleeding or history of cerebral bleeding
- Allergy to ingredients included in either investigational or reference products
- Operated in the head or in the heart

Physical function and activity

- Immobile (defined as the inability to participate in all study-related procedures)
- Being highly physically active, competing as a master athlete and/or partaking in physical demanding training more than 4 times per week, extreme exercising

Medicines and supplements

- Systemic use of antibiotics and/or steroid medication in the last 4 months prior to inclusion
- Use of any nonsteroidal anti-inflammatory drug (NSAID) more than 3 times a week in the last 2 months prior to inclusion
- Use of statins
- Consumption of any NSAID within 7 days of study start
- Regular use, for more than 3 times a week for the last 2 months and/or 7 days prior to inclusion, of medications which according to the principal investigators (ANH and CB) can have an anti-inflammatory effect or affect in any way the intestinal barrier function or have an impact on the study analysis (eg, laxatives, antidiarrheal, and anticholinergic)
- After being included in the study, starting any medication or treatment that could potentially influence the study participation and/or study analysis
- Use of probiotics, prebiotics, fermented foods, kombucha, and any other product known to modulate gut microbiota composition in the last 2 months prior to inclusion

Foreign bodies

- In operated apparatus (eg, pacemaker)
- Aneurysm clips or shunts in the head
- Grenade-splinter or metal-splinter in the body (eg, eyes)
- Metal or electrodes in the body (eg, temp-catheter, aorta stent, and cochlea implant)
- Comprehensive tooth-implants or prosthesis
- Swallowed a video-capsule

Other

- Vegetarian diet
- Claustrophobia
- Left-handed
- Vision problems outside of the range –5 to +3
- Any other reason the investigator feels the participant is not suitable for participation in this aspect of the study
- Regular smoking, use of snuff, nicotine, or e-cigarette use
- Drinking more than 9 standard cups of alcohol per week and/or more than 3 standard cups of alcohol per occasion

### Recruitment

A sample of 114 (38 per arm) community-dwelling men and women, 70 years and older of age, will be recruited in Örebro and surrounding areas through newspaper advertisement and advertisements at primary care facilities, local senior homes, and via local senior organizations. Participants from similar previous studies conducted by our research group (Provita: NCT04126330, AnaBio: NCT05801042) will also be contacted and invited to participate. When interested, prospective participants will be screened for eligibility according to the predetermined inclusion and exclusion criteria, as listed in Textbox 1. Potentially eligible participants will then receive oral and written information from the research team regarding the study, associated benefits and risks, and data management. Before participation in the study, written informed consent will be obtained, and participants will be informed regarding their right to withdraw at any moment during the study. Relevant medical history, concomitant medication, and other demographic data (eg, age and sex) will be recorded in a case report form (CRF). A potential participant will only be included when all inclusion criteria are met, and all exclusion criteria are absent.

### Randomization and Allocation

#### Sequence Generation

Randomization using block allocation sizes of 6 and 3 will be performed before the start of the study in R using the *RandomizeR* package to generate a randomization list. Included participants will be randomized 1:1:1 into 1 of the 3 study groups.

#### Concealment Mechanism

A researcher not involved in the study will assign the participants to the intervention groups and will maintain control of the allocation key in a sealed envelope. This key will not be opened until all preprocessing steps for the functional magnetic resonance imaging (fMRI) analysis (the primary outcome) have been completed.

#### Implementation

Randomization will be performed before the start of the study (baseline), using RStudio (Posit) and performed by an independent statistician, without influence from the investigators or any other associated researcher or collaborator.

### Blinding

All involved researchers will be blind to the initial group allocation. However, blinding for the participants allocated to the no-intervention group will not be possible, as no study products will be distributed to them. The 2 intervention arms, however, will remain blinded in terms of allocation for both researchers and participants. Participants included in the intervention group will receive the MiniMeal products, the reference or control group will receive the reference products, and the no-intervention group will receive no study products and will be instructed to maintain their habitual diet. The no-intervention group will therefore not be blinded to group allocation. This may introduce potential differences between groups related to study engagement, expectations, motivation, or behavioral changes during the study period, which will be considered when interpreting comparisons involving the no-intervention group.

An emergency unblinding procedure will be established to allow the investigators (ANH and CB) the option of disclosing a product assigned to any participant if clinical circumstances require such unblinding. Unblinding will be performed by the principal investigators (ANH and CB) if there is a need, for example, to make a medical decision that requires knowledge about the treatment received. Furthermore, if the study participant experiences any kind of serious adverse event (AE) that she or he attributes to the treatment, even if it is just a perception, we will discontinue the study for that participant. In case of disclosure and unblinding of dietary intervention, the participant will be asked to withdraw from further participation in the trial so as to not bias further analysis (ie, not being blind to the study group can affect the analyses unconsciously).

### Intervention

#### Explanation for the Choice of Comparators

This study will compare the efficacy of an oat- and chickpea-based between-meal study product and a reference or control product to impact older adults. The control study product will be more similar in nutritional content to between-meal products that are commercially available. The no-intervention group will be used to compare the intervention group to the typical, habitual diet of older adults.

#### Intervention Description

Participants in the intervention and the reference or control group will be instructed to consume 2 study products (200 mL) per day to replace or add on to their morning, afternoon, or evening between-meals snack but to keep other dietary habits unchanged. The no-intervention group will maintain their habitual diet. All participants are asked to keep lifestyle and medication stable throughout the study.

During the visits, the participants’ compliance will be checked at weeks 3, 6, and 9, and potential barriers and obstacles interfering with adherence to the study will be addressed. Both intervention products and reference products, that is, soups and fruit drinks, used in this study have been developed by Glucanova AB. The products will be packed in identical, neutral packages, allowing for blinding of the intervention group and the reference product group. The intervention products will be a 200-mL tomato soup and a 200-mL mango or passion drink based on bioprocessed (controlled enzymatic process) oat bran and chickpeas, fruits or vegetables, and natural flavors. The intervention products provide higher amounts of protein, dietary fiber, and energy than the reference products and are based on bioprocessed oat bran and chickpeas. They conform to the Nordic Nutrition Recommendations (NNR 2023) and fulfill European Food Safety Authority nutritional claims regarding fiber, protein, and saturated fat content. Since this study aims to investigate whether there will be differences in health outcomes depending on the nutritional content of between-meal products, the reference and intervention products are not isocaloric. Instead, the content of energy, protein, dietary fiber, and fat is lower in the reference products compared to the intervention products (see details in Table 1). The energy content of the reference products is close in range to an average of similar soups and fruit drinks available on the Swedish market. Instead of oat bran and chickpeas, reference products are based on polished rice flour and corn starch, and the fruits or vegetables are added at lower concentrations than in the intervention products. Table 1 shows the nutritional content of the study products.

**Table 1:** Nutritional composition of the intervention and reference products used in the MiniMeal randomized controlled trial, presented per 100 g.

	Mango or passion drink		Tomato soup	
	Intervention, nutritional values (per 100 g)	Reference, nutritional values (per 100 g)	Intervention, nutritional values (per 100 g)	Reference, nutritional values (per 100 g)
Energy (kcal)	67	48	74	44
Protein	2.5	0.1	4.3	0.4
Carbohydrates	8.6	11.1	6.8	6.9
Carbohydrates of which sugars	5.7	6.8	4.2	2.6
Fiber	1.4	0.1	1.5	0.1
Fiber of which β-glucan	0.7	0	0.5	0
Fat	2	0.21	2.7	1.6
Fat of which saturated	0.2	0.03	0.3	0.2
Salt	0	0.01	0.7	0.8

#### Criteria for Discontinuing or Modifying Allocated Interventions

Based on the ingredients and nutritional content, as well as the included outcomes, serious adverse reactions are not expected. However, in case of any serious AE experienced by a participant, study participation will be discontinued. The severity and relevance of the AEs will be determined as follows:

- Mild: Transient symptoms and no interference with the participant’s daily activities.
- Moderate: Distinct symptoms and moderate interference with the participant’s daily activities but still acceptable.
- Serious: Significant interference with the participant’s daily activities, and unacceptable.

Any AEs during the study will be documented. Participants will also be free to discontinue participation in the study at any stage without needing to state the reason.

#### Strategies to Improve Adherence to Interventions

Each participant randomized to a study product group will receive a logbook and will be instructed to record their daily product consumption. All logbooks will be checked during each study visit, and issues with compliance will be discussed.

#### Relevant Concomitant Care Permitted or Prohibited During the Trial

As consumption of nonsteroid anti-inflammatory drugs is part of the exclusion criteria, participants will be permitted to take paracetamol (acetaminophen) instead.

#### Provisions for Posttrial Care

In case of AEs, participants will be advised to seek appropriate medical assistance if this seems indicated. Any diagnosis, sign, or symptom will be recorded, and the medical doctor in our study team will be consulted.

### Outcomes

#### Primary Outcomes

The primary outcome is to determine whether a 9-week dietary intervention involving daily consumption of nutritionally balanced between-meals based on bioprocessed oat bran and chickpeas alters functional brain activity, assessed using fMRI during the n-back task (a validated paradigm for evaluating working memory performance), compared to a reference or control product.

#### Secondary Outcomes

Secondary outcomes include comparisons between each intervention group (intervention and reference or control) and the nonintervention group to explore broader effects of the intervention on physiological and psychological health. These outcomes comprise a range of predefined health indicators (Textbox 2).

**Primary, secondary, exploratory, and baseline measures in the MiniMeal randomized controlled trial.**

**Primary outcome**

- Functional brain activity (as measured by functional magnetic resonance imaging [fMRI]) during the n-back task

**Secondary outcomes**

Blood markers

- Fasting blood glucose, homeostatic model assessment for insulin resistance index
- Blood lipid status
- Inflammatory markers (eg, high-sensitivity C-reactive protein, interleukin-6, tumor necrosis factor, and interferon-γ)
- Immune cell characterization and stimulation
- Intestinal barrier markers (intestinal fatty-acid binding protein, zonulin, etc)

Self-reported measures of health, well-being, physical activity, and function

- Perceived Stress Scale
- Hospital Anxiety and Depression Scale
- 36-Item Short Form survey
- Western Ontario and McMaster Osteoarthritis Index
- Clinical Outcomes in Routine Evaluation General Population
- Gastrointestinal Symptoms Rating Scale
- The Pittsburgh Sleep Quality Index
- International Physical Activity Questionnaire for the Elderly

Cognitive function

- Off-line multidomain cognitive test battery
- Computer-based, word recognition task

Neuroimaging

- Resting state functional connectivity as measured by resting state fMRI
- Brain morphology from structural scans
- Functional connectivity during the n-back task

Body composition

- Bioelectrical impedance
- BMI
- Waist circumference
- Thigh and lower leg muscle volume (caliper)

Physical function

- Aerobic capacity (walk test on treadmill)
- Balance (single leg stance balance test)
- Muscle performance (30-second repeated chair raise, leg strength, and handgrip strength)

**Exploratory outcomes**

- Microbiota composition (feces)
- Metabolomics (feces)
- Markers of oxidative stress (H_2_O_2_; blood)
- Epigenetics (blood)
- Extracellular vesicles (blood)
- Micro-RNA (blood)
- Analysis of neuro-related blood markers (eg, brain-derived neurotrophic factor and serotonin)
- Dietary intake (Food Frequency Questionnaire [FFQ] after the intervention to assess whether participants have altered dietary patterns during the intervention)

**Measurements to characterize the study population**

- Microbiota composition (feces)
- Dietary intake (Meal-Q FFQ)
- Past and present physical activity behaviors (Historical Adulthood Physical Activity Questionnaire and accelerometer)
- Cognitive state (Montreal Cognitive Assessment)

### Assessment Schedule

Participants will come to the study center on 7 occasions, 1 time before (screening visit) and 6 times during the study period to perform 1 or more tests and/or examinations. After the screening, visits 1 and 2 will take place at baseline. Visits 3 and 4 will be the mid-study visits in weeks 3 and 6 of the interventional period. Visits 5 and 6 will take place in the last interventional week, that is, week 9. A timeline of study visits can be found in Figure 1, and a SPIRIT schedule of enrollment, interventions, and assessments can be found in Figure 2.

**Figure 1:**
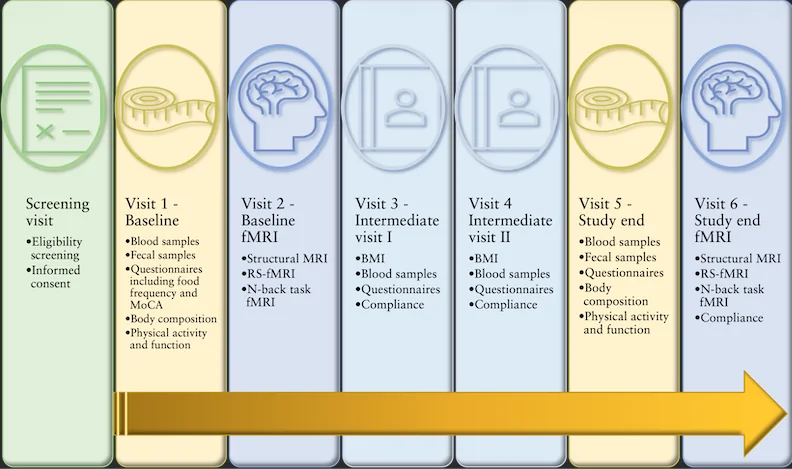
Timeline of study visits. Overview of the MiniMeal study visits and assessments. Participants complete a screening visit followed by 6 study visits over the 9-week intervention period. Baseline and end-of-study assessments include biological sampling, body composition, physical and cognitive function, questionnaires, and magnetic resonance imaging, while intermediate visits are conducted to monitor compliance and collect selected outcome measures. fMRI: functional magnetic resonance imaging; MoCA: Montreal Cognitive Assessment; RS-fMRI: resting state functional magnetic resonance imaging.

**Figure 2:**
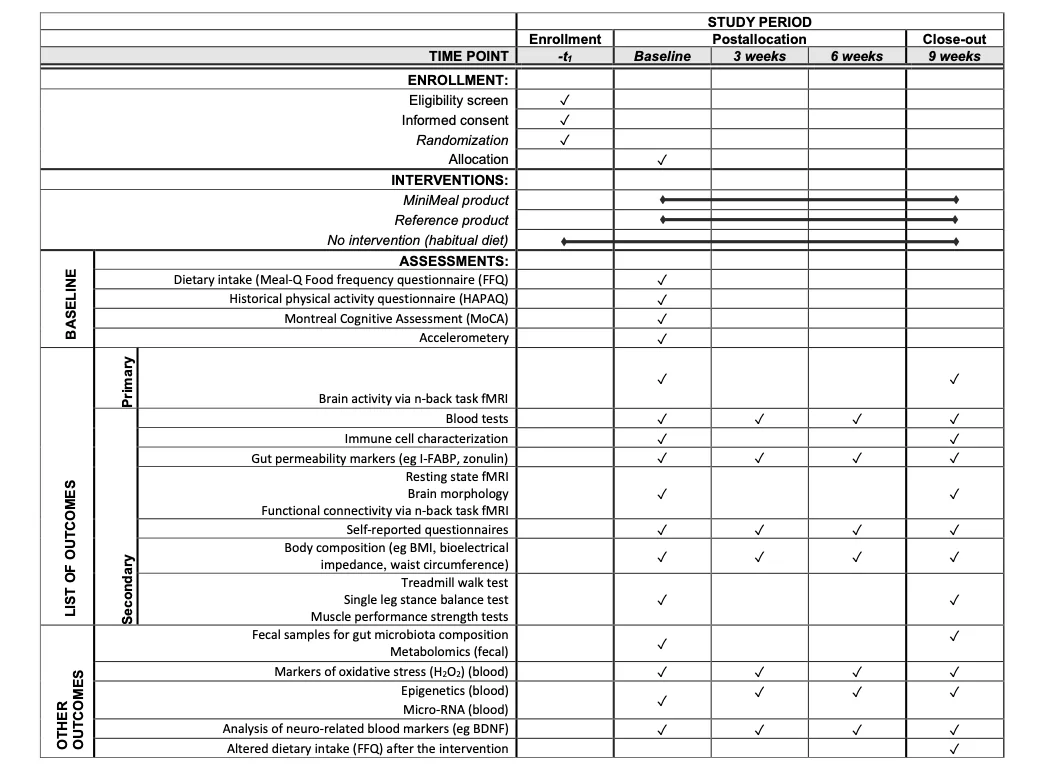
SPIRIT schedule of enrollment, interventions, and assessments in the MiniMeal randomized controlled trial. The schedule shows the timing of enrollment, allocation, the 9-week MiniMeal, reference product, and no-intervention conditions, and primary, secondary, exploratory, and baseline assessments. ✓ indicates the time point at which each assessment is performed. BDNF: brain-derived neurotrophic factor; fMRI: functional magnetic resonance imaging; I-FABP: intestinal fatty-acid binding protein; SPIRIT: Standard Protocol Items: Recommendations for Interventional Trials.

### Sample Size

Although the number of studies using magnetic resonance imaging (MRI) to assess the effects of food products on brain activity during a cognitive task in older populations is limited, a few studies can aid in determining an adequate sample size. Boespflug et al [41] conducted a study to examine the effects of fish oil supplementation on older adults (62-80 years) with subjective cognitive impairment and were able to detect differences in blood-oxygen-level-dependent (BOLD) activation during an n-back task with 11 (fish oil) and 10 (placebo) participants in each group. Boespflug et al [42] also conducted a study with 16 older adults to examine the effects of a food supplement on a working memory task, performing the n-back task in healthy older adults. From this sample size, they were able to detect education by age interactions for task-related activity.

A very limited number of studies have used fMRI to assess the effects of food products in older adults [41,42], thus making the determination of an adequate sample size rather difficult. For the primary outcome, the intervention and reference arms will be compared regarding changes in BOLD signal in approximately 20 regions of the brain (implicated in cognitive function and the GBA), with the aim to be able to detect a change with Cohen *d*=delta/SD=1 (delta difference in mean values between the 2 groups) with 80% power. Counting in a maximum of 10% dropouts, based on a 2-sample *t* test and working with a Bonferroni-adjusted significance level of 0.05/20, this would require approximately n=36 per group. To account for additional sources of variability inherent to fMRI data in older populations—including motion artifacts, physiological noise, potential nonnormality of BOLD responses, and the possibility of unusable scans—the sample size was conservatively inflated by an additional 5%. Thus, 38 participants will be recruited per arm.

### Data Collection

#### Plans for Assessment and Collection of Outcomes

Primary, secondary, and exploratory end points are collected as outlined below.

#### Blood Samples (Visits 1, 3, 4, and 5)

Blood samples of approximately 30 mL will be collected according to good clinical practice (GCP) and good laboratory practice (GLP) at Örebro University Hospital (CUSÖ) and will be used to assess systemic inflammation, glucose, insulin, blood fats, intestinal function markers, and immunological markers, as part of the secondary and exploratory outcomes. Systolic and diastolic blood pressure will be measured, and the mean of 2 successive measurements will be used.

#### Fecal Samples (Visits 1 and 5)

To assess microbial composition and fecal metabolites, fecal samples will be collected at home by the participants. The fecal samples will be stored at home at –20 °C and transferred in the provided appropriate transport container to the study site. Once there, the samples will be stored at –80 °C until analysis.

#### fMRI-Based Assessments (Visits 2 and 6)

MRI assessment is a safe method that has been extensively used for clinical and research purposes during the last 30 years. All MRI examinations will be performed by a fully trained MRI technician or nurse and a qualified investigator, who will conduct the MRI session and be present at all times. Acquisitions will be based on a routine MRI procedure of only approved sequences for research and clinical purposes that do not require any contrast agent. Ionizing radiation is not involved, and the procedure is believed to be without significant risk to patients. Participants will be screened for MRI contraindications and will be required to fill in a safety-check form each time, before entering the scanner.

Within 3 days of visit 1 at baseline and visit 5 at study end, participants will visit the CEBIO to perform the MRI and computer-based cognitive test (visits 2 and 6). For both MRI visits, the participants will be asked to perform the same routines on both days, including avoidance of physical exercise and limiting the consumption of caffeine to 1 cup of coffee or tea in the morning.

The principal investigator will keep project records confidential. Examined data will be archived in digital format on media retained by the principal investigator. Image data transfers, which are required for fMRI postprocessing, will be pseudoanonymized, and scanner/examination/series/image numbers will only be used.

A 3.0T MR system (GE HealthCare) will be used. Foam pads will provide comfort and lead to mild immobilization of the head, as participants will be required to lie still inside the MRI unit for approximately a total of 40 minutes. An initial structural scan (T1-weighted) of about 4.5 minutes will be performed to compensate for the lower spatial resolution of the resting state fMRI and task-based fMRI acquisitions that follow. Following the structural scan, a resting state of the brain with a duration of 10 minutes will be acquired. The participants will be asked to keep their eyes open during the entire test and fixate on a cross sign displayed on a screen during the resting-state scan (eg, to avoid falling asleep).

After the resting-state scan, participants will receive instructions to perform an fMRI-based n-back task (1-, 2-, and 3-back in random blocks) working memory test [43] to assess the effect of nutritious in-between meals on cognition (primary outcome). This task consists of a sequence of single letters that appear on a screen. For each one of the letters presented, participants will report if the letter currently seen is the same as the letter shown 1, 2, or 3 letters back. Prior to starting the scanning session, participants will practice the task outside of the scanner until the task is well-understood. Performance during the conducted task, brain activity, and functional connectivity will be compared between groups.

#### Assessment of Cognitive Function (Visit 1, Baseline)

The Montreal Cognitive Assessment (MoCA) will be used to characterize the cognitive status of the participant population at baseline (visit 1) [44]. The MoCA assesses short-term memory, visuospatial abilities, executive functions, attention, working memory, language, and orientation to time and space and is a commonly used instrument for detecting cognitive impairments in the clinical setting. MoCA can be used to indicate normal cognitive abilities, mild cognitive impairments, and dementia. The instrument, however, needs to be used together with other evaluations to enable a dementia diagnosis, which is not the purpose of this trial.

#### Assessment of Dietary Behavior (Visits 1 and 5)

Habitual dietary behavior will be assessed and monitored through the Meal-Q online Food Frequency Questionnaire, assessing 174 items. This instrument is validated for use in Sweden and shows fair reproducibility [45]. It provides information regarding the participant’s eating pattern during a certain time period prior to completion of the questionnaire. In this study, the Meal-Q will be used to assess food habits over the past month. The participants completing the trial will also be asked to fill in the questionnaire at visit 1 (baseline) and visit 5 (study end), as well as 2 months after the intervention has ended in order to detect changes in dietary behavior that persist after the intervention and the influence of baseline dietary patterns on the efficacy of the intervention.

#### Anthropometry and Body Composition (Visits 1, 3, 4, and 5)

For anthropometry, body height and weight will be measured using a stadiometer and digital scale, respectively, following standard procedures. Total and regional body composition including fat percentage and lean body mass will be assessed using bioelectrical impedance analysis. BMI and waist circumference will be measured to further evaluate adiposity and cardiometabolic risk. BMI will be calculated as weight (kilograms) divided by height (meters) squared. Waist circumference will be measured at the midpoint between the iliac crest and lower costal margin to the nearest 0.1 cm using a measuring tape.

#### Physical Activity (Visits 1 and 5)

To assess present physical activity (PA) and sedentary behaviors at baseline, participants will wear the GT3x accelerometer (Actigraph) for 1 week. This monitor measures PA in all 3 planes using the arbitrary unit (counts per minute). Participants will be instructed to wear the monitor on their right hip (iliac crest) during all waking hours, except during water activities. They will be asked to record any events when the monitor is removed and note the time on and off for sleep times at night. Daily average times spent in sedentary behavior, light-intensity PA, and moderate-to-vigorous intensity PA will be derived based on previous work [46,47].

Furthermore, PA will also be assessed by the International Physical Activity Questionnaire for the Elderly (IPAQ-E) at baseline and at the end of the study. The IPAQ-E is based on the short form International Physical Activity Questionnaire and modified to be used in older populations. The IPAQ-E has been validated to classify participants aged 65 years and older into PA categories, to rank individuals, or to identify individuals meeting certain PA criteria [48]. The questionnaire assesses PA over the last 7 days and comprises 4 questions.

To evaluate participants’ past PA behaviors between the ages of 20 and 65 years, the Historical Adulthood Physical Activity Questionnaire (HAPAQ) will be used. The HAPAQ has been validated as a reliable tool for ranking individuals based on their previous PA behaviors, as compared to objective PA assessments [49]. This questionnaire assesses past leisure time and occupational PA and comprises 14 questions.

#### Physical Function (Visits 1 and 5)

The participants’ physical function will be evaluated through a battery of tests, which assesses their balance, muscle strength, and cardiorespiratory fitness. Balance performance will be assessed using the single leg stance test, where participants stand barefoot on their dominant leg with arms crossed and eyes open and closed [50]. The ability to repeatedly transfer from a seated to standing position will be assessed using the validated 30-second chair stand test [51], where participants will be asked to rise from a standard chair without armrests as quickly as possible with their arms folded across their chests for 30 seconds. Upper body strength will be measured using a standardized procedure using a handheld dynamometer. To assess lower limb muscle function during weight-bearing multijoint conditions, participants will perform a one-repetition maximum by using a valid and reliable measure of dynamic and/or isometric strength. The one-repetition maximum is considered the gold standard for the assessment of maximal strength and is a safe and practical test to perform in older populations with the right support and instruction [52]. Cardiorespiratory fitness will be assessed using the modified Bruce Treadmill Walk test, which is validated for use in older populations and populations with a lower functional capacity [53].

#### Assessment of Sleep (Visits 1, 3, 4, and 5)

To assess sleep quality and sleep disturbances over a predefined interval, the Pittsburgh Sleep Quality Index will be used [54]. This questionnaire captures sleep in the following areas: subjective sleep quality, sleep latency, sleep duration, habitual sleep efficiency, sleep disturbances, use of sleeping medication, and daytime dysfunction.

#### Assessment of Quality of Life, Well-Being, Stress, General Health Status, and Osteoarthritis (Visits 1, 3, 4, and 5)

To evaluate psychological well-being, the translated and validated Swedish version of the Clinical Outcomes in Routine Evaluation General Population questionnaire will be used [55]. The widely used and validated Hospital Anxiety and Depression Scale will be used to assess symptoms of depression and anxiety [56]. To measure the perception of stress, we will use the Perceived Stress Scale [57]. The Perceived Stress Scale measures to which degree life is perceived as stressful. To assess general health status, the Short Form Health Survey 36 items (SF-36) health survey will be used [58]. The SF-36 captures 8 dimensions of health [59]. In addition, the Knee Injury and Osteoarthritis Outcome Score, a widely used questionnaire to evaluate knee injury and osteoarthritis, will be used. This instrument assesses 5 outcomes (pain, other symptoms, activities of daily living, sport and recreation function, and knee-related quality of life) [60] and has previously been used in the randomized controlled trial setting [61]. Participants are requested to fill in all questionnaires 1 or 2 days prior to the visits.

#### Gastrointestinal Symptoms (Visits 1, 3, 4, and 5)

To assess gastrointestinal symptoms, the well-validated Gastrointestinal Symptoms Rating Scale will be used. The Gastrointestinal Symptoms Rating Scale covers 15 items, including acid reflux, hunger pains, rumbling, bloating, burping, gas, constipation, and diarrhea, that are rated on a scale from no discomfort at all to very severe discomfort [62].

#### Plans to Promote Participant Retention and Complete Follow-Up

Prior to the start of the study, participants are thoroughly instructed regarding the study procedures, and extra information or assistance is offered when needed. At each study visit, participants are checked on study compliance, barriers for completion of the study are identified, and solutions are offered.

### Data Management

All data will be collected using a CRF. The investigators will capture every part of the clinical data in the CRF. It is the responsibility of the investigators to maintain accurate CRFs to record all observations and data pertinent to the investigation. For instance, when information needs to be modified, the correction should be made, and the original information to be modified should not be erased but overwritten in a way that both the old information and the newly added one are visible. The corrected information will be transcribed by the authorized person next to the previous value, signed or initialed, and dated. Relevant parts of the CRF will be entered into a database, and all physically recorded data will be stored securely in places with limited access. Data management will be in accordance with the Data Management Plan of the School of Medical Sciences at Örebro University. Data will be stored securely at Örebro University servers and only accessible inside Örebro University or through virtual private network from outside the university. Proper folder structure with appropriate read and write access to the data will be used to store project data. Access rights to the project folders will be based on a user’s role within the project.

### Statistical Analyses

#### Statistical Methods for Primary and Secondary Outcomes

Analysis of the primary outcome will be performed in SPM (version 25; Wellcome Centre for Human Neuroimaging, University College London) [63]. The primary, hypothesis-driven analyses will consist of region-of-interest (ROI) analyses focusing on predefined brain regions selected a priori based on the existing literature and previous studies in our research group. In addition to the primary ROI analyses, secondary whole-brain voxel-wise analyses will be conducted to identify activation changes outside the predefined ROIs. Multiple comparisons will be controlled using false discovery rate correction with SPM, and false discovery rate–corrected *P* values (*P*FDR<.05) will be considered significant. Effect sizes and corresponding CIs will be reported alongside statistical significance to facilitate interpretation of the magnitude and precision of intervention effects, including findings that do not reach statistical significance.

Preprocessing will be performed using CONN [64] (RRID:SCR009550) release 25.b [65] and SPM (RRID:SCR_007037) release 12.7771 [66]. Preprocessing will follow the standard pipeline in CONN, which includes realignment, slice timing correction, outlier detection, coregistration, segmentation, normalization, and smoothing (8 mm full width at half maximum).

Load-dependent brain activation will be assessed using the predefined task contrasts: 3-back>1-back and 2-back>1-back. Each contrast will be set up as a general linear model in SPM using a flexible factorial design with subject, time, and group as factors. Group differences in change in load-dependent activation will be evaluated using group×time interaction. Predefined pairwise comparisons (A vs B, A vs C, and B vs C) will be examined separately for each primary or predefined contrast.

For non-fMRI–related outcomes, data will be checked for normal distribution using appropriate tests as well as visual inspection. Depending on normality of the data, suitable parametric or nonparametric tests will be used. All remaining statistical tests will be performed using R (R Foundation for Statistical Computing), SPSS (IBM Corp), SPM12, GraphPad Prism (GraphPad Software), or sent out to specialized companies (eg, Food Frequency Questionnaire) depending on the specific type of analysis. Investigators can decide to use other statistical software if considered more appropriate or convenient.

Per-protocol analyses will serve as the primary analytical approach for evaluating intervention effects, as the primary objective of this study is to assess the physiological and neurobiological effects of the intervention among participants who adhered to the study protocol. Intention-to-treat analyses, including all randomized participants according to their assigned treatment group, will also be conducted as sensitivity analyses to evaluate the robustness of the findings.

Given the broad range of secondary outcomes, multiple testing adjustments will be applied within related families of outcomes (eg, neuroimaging, inflammatory biomarkers, and metabolic markers) where appropriate. Results from secondary outcomes will be interpreted as supportive, while findings from exploratory, subgroup, responder versus nonresponder, and other post hoc analyses will be considered hypothesis-generating and interpreted cautiously.

In addition to evaluating the independent effects of the intervention on the primary neuroimaging outcome and each secondary outcome, exploratory analyses will examine associations between changes in task-related brain activation and changes in gut health markers, circulating inflammatory and metabolic biomarkers, and physical function or activity measures. These complementary analyses are intended to provide insight into potential biological mechanisms underlying any observed intervention effects and to generate hypotheses regarding interactions within the GBA. While the study is not powered or specifically designed to formally establish causal pathways or conduct confirmatory mediation analyses, the simultaneous assessment of these outcomes will allow the exploration of how changes across multiple biological systems may relate to changes in brain function.

#### Interim Analyses

No interim analysis is planned for this study.

#### Methods for Additional Analyses (eg, Subgroup Analyses)

Additional prespecified covariates (eg, age, sex, or baseline values) may be included depending on the outcome measure and scientific rationale. Due to the exploratory nature of this study, post hoc or cross-sectional analyses based on, for example, various subgroups may also be carried out. In addition, we plan to characterize participants as responders versus nonresponders to the intervention, as this may help to advance personalization and precision in future dietary interventions.

#### Methods in Analysis to Handle Protocol Nonadherence and Any Statistical Methods to Handle Missing Data

If a participant fails to meet the predefined qualifications for compliance or violates study protocol, this participant will not be included in the analysis of study outcomes. Mixed-effects models will be used for longitudinal outcomes whenever appropriate, allowing inclusion of participants with incomplete follow-up data under the assumption that data are missing at random. The extent and pattern of missing data will be examined, and additional sensitivity analyses may be conducted if warranted.

#### Plans to Give Access to the Full Protocol, Participant-Level Data, and Statistical Code

According to Swedish ethics regulations, the raw data cannot be shared without an approved ethics application from the National Swedish Ethics Authority. An ethical permit can only be obtained for research being conducted within Sweden. Data can be requested as a public document via written request to the study team and will have to undergo a confidentiality assessment to determine what can be released.

### Monitoring

#### Management and Oversight

The current project will be managed by principal investigators CB and ANH and conducted within the Nutrition-Gut-Brain Interactions (NGBI) Research Centre at Örebro University. The principal investigators are responsible for the planning and performance of the trial. The study takes place at Örebro University.

#### Composition of the Coordinating Center and Trial Steering Committee

We will conduct this trial without establishing a trial steering committee. This is a single-center study where the coordinating center comprises members of the NGBI Research Center at Örebro University, and all study visits will be conducted by the study team, alongside hospital staff to assist with obtaining blood samples and overseeing the MRI. The study staff will meet once a month to discuss the trial progression.

#### Composition of the Data Monitoring Committee, Its Role, and Reporting Structure

A data monitoring committee will not be established. Instead, monitoring will be conducted within the research group by personnel designated by the principal investigator, using a mutual monitoring system. Monitoring will be performed by research members other than those responsible for obtaining participant explanations and consent.

#### AE Reporting and Harms

Due to the benign intervention and the study team’s previous familiarity with all procedures and assessments, no adverse effects among the participants are expected. However, all adversities will be recorded, and the participants will be advised to seek appropriate medical assistance if this seems indicated. Any diagnosis, sign, or symptom will be recorded, and the medical doctor in our team will be consulted. In case of any serious AE experienced by a participant, study participation will be discontinued. Participants are also free to discontinue participation in the study at any stage without stating the reason. All tests conducted will be performed by qualified and competent personnel.

#### Frequency and Plans for Auditing Trial Conduct

Not applicable.

### Ethical Considerations

The MiniMeal study has been approved by the Swedish Ethical Review Authority (identification number (Dnr) 2023-07609-01) and registered at ClinicalTrials.gov (NCT06353984). All participation in the study is voluntary, and all procedures will be conducted according to fundamental ethical principles as outlined in the Declaration of Helsinki (World Medical Association Declaration of Helsinki) and GCPs in Europe (European Medicines Agency, Science Medicines Health), as well as GLPs. The safety, health, well-being, and anonymity of the participants will be prioritized. Prior to the study, participants will provide written informed consent regarding participation and publication of study data. Any important protocol modifications will be subject to an amendment to the Swedish Ethical Review Authority for new approval. Informed consent will be taken by an authorized member of the study team. In case of an amendment in the research protocol, approval will be obtained from the Swedish Ethical Review Authority (Etikprövningsmyndigheten). Collection and preparation of all laboratory samples at the clinical center will be performed in a standardized way according to a written laboratory manual, to GCP and GLP, all ensuring the quality of the procedures. Samples will be stored at the Örebro Hospital Biobank, and all the procedures will be following the Guide to Biobanks in Sweden—Access to Samples for Research and Clinical Trails (Biobank Sverige)—and adhere to the Swedish Biobank in Medical Care Act (SFS 2002:297), of which, all potential participants will be informed in the participant information. All participants will be assigned a unique study ID code, which will be documented and protected by the principal investigator according to good research practice guidelines. Data will be pseudonymized and stored under the assigned study ID code. If necessary, the research team will have access to the code list, but no personal details of participants will be disclosed in any forthcoming publications. Personal information will be anonymized using a participant number. All data will be stored in secure cabinets and backed up on an electronic system with restricted access. Dissemination of findings will take place through presentations or posters at national and international conferences and peer-reviewed publications in high-quality scientific journals, as well as popular science dissemination in regional and national newspapers and outreach activities such as local presentations. All participants receive US $209 after completion of the study.

## Results

The study protocol (version 1) received ethics approval from the Swedish Ethical Review Authority (Etikprövningsmyndigheten; dnr 2023-07609-01) and was registered at ClinicalTrials.gov (NCT06353984). Recruitment started on August 8, 2024, and was completed on December 18, 2025. Data collection started on August 26, 2024, and was completed on February 27, 2026. Data analysis is ongoing since January 7, 2026. A total of 114 participants were enrolled, of whom, 106 completed all 6 study visits. An overview of the study design and participant flow is shown in Figure 3.

**Figure 3:**
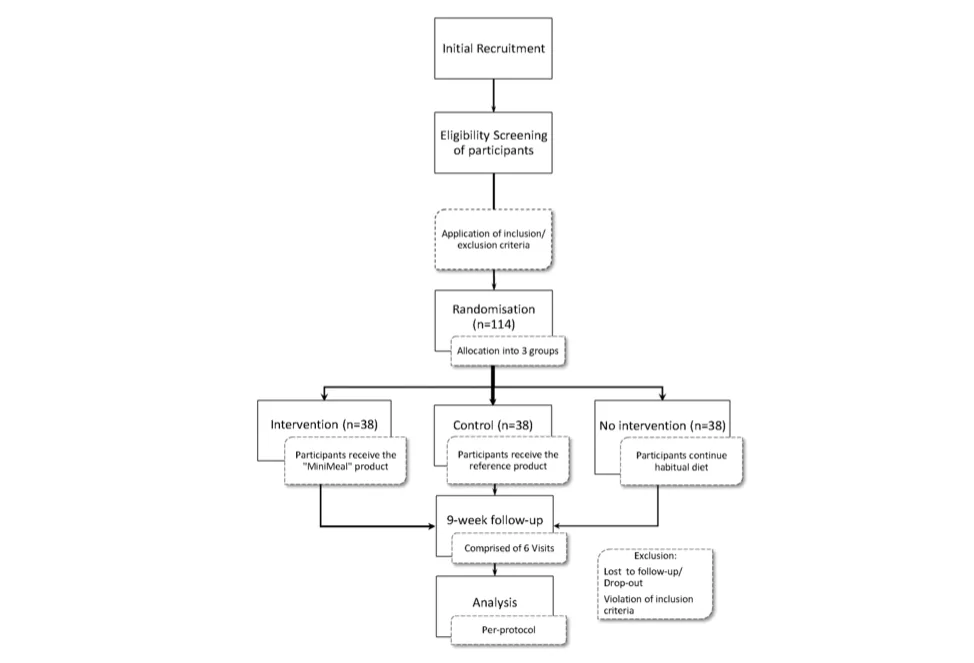
Overview of the MiniMeal study design and participant flow. A total of 114 eligible participants were randomized in a 1:1:1 ratio to the MiniMeal intervention group (n=38), reference or control group (n=38), or no-intervention group (n=38). Participants in the intervention and reference or control groups received their respective study products, whereas participants in the no-intervention group continued their habitual diet. All groups underwent a 9-week intervention period comprising 6 study visits, followed by outcome assessment.

## Discussion

### Principal Findings

The primary aim of this study is to investigate whether a nutritionally balanced between-meal product can improve functional brain activity during a cognitive task in healthy older adults. We hypothesize that the 9-week dietary intervention will affect task-related brain activation within predefined regions associated with working memory. In addition, we anticipate that the intervention may positively impact cognitive performance, inflammatory markers, gut function, and physical function, thus impacting several aspects of the GBA. By combining neuroimaging with comprehensive assessments of cognition, gut health, inflammation, and physical function, this study aims to provide novel insights into the mechanism linking nutrition and brain health in older adults.

### Comparison With Previous Studies

Given the negative impact of cognitive impairment on older adults’ health and well-being, as well as the increasing demand regarding medical care and associated costs, the primary aim of this study is to investigate the potential benefits of a nutritionally balanced between-meal product on functional brain activity during a cognitive task in older adults. An earlier cross-sectional study by Damoiseaux et al [13] investigated whether functional connectivity of intrinsic brain activity in the DMN is affected by normal aging and found decreased activity in older compared to younger participants. Persson et al [12] investigated the longitudinal changes in DMN while performing a memory task and found a reduced deactivation in DMN regions during advancing age. At the same time, there is evidence from randomized controlled trials that a culturally adapted and individually tailored diet can slow down cognitive aging [67]. Furthermore, evidence indicates that the Mediterranean diet can contribute to improved cognitive function [68].

These previous studies have provided some novel insights into DMN function, as well as linking such measures to systemic inflammation and the importance of a healthy diet. However, to our knowledge, no randomized controlled trials have been conducted to establish any pathophysiological associations. Hence, we aim to measure functional brain activity during a cognitive task by fMRI before and after a 9-week dietary intervention that we hypothesize will positively impact several aspects of the GBA.

As dietary modifications have the potential to modulate both gut microbiota composition and systemic inflammation, they are an attractive option for addressing the aforementioned research questions. However, making long-term, sustainable dietary alterations in older adults proves challenging, given the tendency to revert to former dietary habits over time [69]. Consequently, an alternative strategy involving the addition of healthy between-meal products emerges as an interesting and potentially more sustainable solution for enhancing both the quantitative and qualitative aspects of dietary intake among older adults. Thus, this approach could offer incremental improvements in dietary habits without necessitating drastic changes, thereby promoting long-term adherence and optimizing health outcomes in this population.

The MiniMeal products used in this study contain oat bran and chickpeas, 2 natural food components that have the potential to positively affect gut microbiota and lower inflammation. These 2 areas are of particular interest as they can promote health in older adults including improved brain function. Dietary fibers such as β-glucans in oats, oligosaccharides such as ciceritol in chickpea grains, and the chickpea isoflavone Biochanin A present significant neuroprotective capacity based on preclinical evidence [40,70,71]. Furthermore, intake of additional dietary fiber derived from various sources (grains, legumes, and vegetables or fruits) in the study products has been associated with improved cognitive function and healthy aging [72]. Additionally, the fermentation of these very ingredients in the gut could improve the production of microbial metabolites such as short-chain fatty acids, which may cross the blood-brain barrier and exert their antioxidant and anti-inflammatory properties on the brain with the potential to prevent neurodegeneration [73]. Simultaneously, the extra protein and increased caloric intake also have the potential to prevent malnutrition in individuals at risk and decrease the impact of anabolic resistance [74]. Improving protein synthesis is of particular importance for a healthy bone and muscular system in older individuals. As such, extra caloric intake combined with protein can be seen as an important strategy to fend off frailty [75].

### Strengths and Limitations

An important strength of the present study is the comprehensive characterization of healthy older adults through multimodal assessments of neuroimaging, cognition, gut health, inflammatory biomarkers, dietary intake, gastrointestinal function, physical function, PA, sleep, and well-being. This broad approach acknowledges the complex interactions within the GBA and may provide valuable mechanistic insight into the pathways through which dietary interventions influence brain health. The inclusion of several secondary and exploratory outcomes is intended to complement the primary neuroimaging outcome and generate hypotheses for future studies while facilitating comparison of the study population with other cohorts.

Nonetheless, designing such a protocol is associated with several challenges. There is currently no universally accepted approach for sample size calculations in functional MRI studies, although considerable methodological advances have been made in recent years [76]. The sample size for the present study was determined based on fMRI-specific calculations together with previous experience from similar studies including adults (aged 18-80 years), as described in the Sample Size and Statistical Analyses sections. Furthermore, although the inclusion of multiple secondary and exploratory outcomes provides a comprehensive assessment of healthy aging, it also increases the complexity of the statistical analyses. To address this, primary, secondary, and exploratory outcomes have been predefined, and appropriate adjustments for multiple comparisons are described in the Statistical Analyses section.

In addition, although task-based fMRI provides a sensitive measure of changes in functional brain activity, there is currently limited evidence regarding the magnitude of BOLD signal changes that should be considered biologically meaningful following nutritional interventions. Given the expected variability of fMRI measures and the relatively short intervention duration, the present study is primarily designed to detect intervention-associated changes and provide estimates of effect sizes rather than establish clinically meaningful thresholds.

Another important consideration is the expected frequency of incidental findings in this study population. As participants aged 70 years and older are included, incidental findings are expected in approximately 5%-20% of MRI scans and around 10% of blood samples [6-8], although fewer than 2% are anticipated to be clinically significant [6]. Brain scans will be acquired by specialized nurses and reviewed by a neuroradiologist. If findings are considered clinically relevant and not attributable to normal aging, participants will be informed and advised to seek further medical evaluation. Prior to enrollment, participants will be informed that the MRI examination is performed for research purposes and is not intended as a clinical screening tool.

Finally, in food intervention trials, the reference product is often designed to be isocaloric to the intervention. In the present study, however, the objective is to determine whether nutritionally balanced between-meal products with higher nutritional quality and energy content than commonly consumed products improve brain health and other indicators of healthy aging. Therefore, the reference soup and drink were designed by Glucanova AB to reflect the average nutritional composition of commonly available tomato soups and fruit drinks on the Swedish market, thereby providing a comparison that more closely reflects real-world dietary choices.

### Future Directions

If the intervention demonstrates favorable effects on functional brain activity and related outcomes, this study will potentially provide important mechanistic evidence supporting the role of nutrition in healthy cognitive aging. The findings could then contribute to the development of larger randomized controlled trials and help identify dietary strategies and participant characteristics associated with improved brain health, thereby supporting more personalized nutritional interventions for older adults.

### Summary

In light of the global increase in cognitive decline among older adults, novel preventive and therapeutic strategies are urgently needed. Greater understanding of both the efficacy of nutritious meals and dietary patterns, and the mechanisms underlying their potential effects on cognitive function, is essential. This paper outlines a randomized controlled trial designed to evaluate the effects of a newly developed, nutritionally balanced between-meal product on functional brain activity during a cognitive task, as the primary outcome. The study is expected to provide new insights into the relationship between dietary habits and cognitive function in older adults and contribute robust evidence from a rigorously designed randomized controlled trial to inform future research. At a societal level, the findings may help clarify the potential of diet-based approaches to support cognitive health and healthy aging in older populations.

## Data Availability

The databases generated or analyzed during this study are not publicly available due to the Swedish Public Access to Information and Security Act but are potentially available from the corresponding author on reasonable request, subject to a review of privacy.

## Abbreviations

AE: adverse event
BOLD: blood oxygenation level dependent
CEBIO: Center for Experimental and Biomedical Imaging in Örebro
CRF: case report form
DMN: default mode network
fMRI: functional magnetic resonance imaging
GBA: gut-brain axis
GCP: good clinical practice
GLP: good laboratory practice
HAPAQ: Historical Adulthood Physical Activity Questionnaire
IPAQ-E: International Physical Activity Questionnaire for the Elderly
MoCA: Montreal Cognitive Assessment
MRI: magnetic resonance imaging
NGBI: Nutrition-Gut-Brain Interactions
PA: physical activity
ROI: region of interest
SF-36: Short Form Health Survey 36 items
